# Classical McIndoe Technique Versus the McIndoe Technique with a Neovaginal PACIENA Prosthesis^®^ and No Skin Graft

**DOI:** 10.3390/jcm9113648

**Published:** 2020-11-13

**Authors:** Victoria Navarro, Maria Isabel Acién, Pedro Acién

**Affiliations:** 1 Obstetrics and Gynecology Service, Elda University Hospital, 03600 Elda, Alicante, Spain; victoria.nl@coma.es; 2 Obstetrics and Gynecology Service, San Juan University Hospital, 03550 San Juan, Alicante, Spain; 3 Department/Division of Gynecology, Miguel Hernández University, Campus of San Juan, 03550 San Juan, Alicante, Spain; acien@umh.es

**Keywords:** vaginal agenesis, vaginoplasty, neovaginal prosthesis, Rokitansky syndrome, PACIENA^®^ prosthesis, modified McIndoe technique

## Abstract

An observational, retrospective study was completed to compare the results of the PACIENA clinical trial (using the modified McIndoe technique) with a historical control group of nine patients who were operated on at San Juan University Hospital (1992–2015) using the classic technique. The PACIENA clinical trial included seven patients with vaginal agenesis who were operated on at two reference sites (May 2017–May 2018) using a neovaginal polylactic acid (PLA) prosthesis (PACIENA^®^ prosthesis) and avoiding the use of a skin graft. The results illustrate a reduction in the length of surgery, 86.43 ± 4.75 min in the group with no skin graft compared to 155.56 ± 28.44 in the control group (*p* < 0.05); and reduction in the length of hospitalization time. Differences were also registered in the length of the neovagina, the average being 8.93 ± 1.42 cm for cases and 6.56 ± 1.13 cm for controls, with no differences in neovaginal epithelialization times or in the satisfaction of sexual relations occurring between groups. The modification of the classical McIndoe technique using the neovaginal PACIENA^®^ prosthesis appears to be successful, obtaining good clinical results with shorter surgery and hospitalization times.

## 1. Introduction

Vaginal agenesis is a type of genital malformation that can be seen in individuals with Rokitansky syndrome (1 in 4500–5000 women) [1], Morris syndrome (1 in 64,000 women), MURCS association (Müllerian duct aplasia, renal aplasia and cervicothoracic somite dysplasia; 1 in 50,000 women), among other syndromes. Several surgical techniques are used to correct vaginal agenesis, with the McIndoe technique being the most common [2].

Acién et al. [3] published a pilot study in which the feasibility and clinical outcomes of vaginoplasty with no skin graft using a polylactic acid, or PLA, prosthesis (PACIENA^®^) and Interceed [4] were assessed. This was a prospective, multicenter study on a medical device (PACIENA^®^ prosthesis) in which seven patients were operated on and there was 6 months of subsequent follow-ups, obtaining good anatomical and functional results. The basis for the results stem from the PACIENA^®^ prosthesis, a vaginal device with an anatomical design allowing urethral protection, which together with its lightness reduces the risk of decubitus ulcers. Furthermore, the prosthesis is made of a biocompatible material with properties related to epithelial healing (PLA), eliminating the need for a skin graft.

The purpose of this paper is to compare the results of the aforementioned study with patients operated on using the classical McIndoe technique with a skin graft at the same site, in order to assess this modified surgical technique.

Study Objective

The objective of this study was to determine whether or not the modified McIndoe surgical technique with no skin graft using a neovaginal PLA prosthesis (PACIENA^®^ prosthesis) facilitates surgery without scarring the donor area of the skin graft while reducing surgery and hospitalization times, registering good functional results and providing patient satisfaction when compared with historical cases who were operated on using the traditional technique.

## 2. Experimental Section

This was a comparative, observational, retrospective study that compared the results of the cases studied in the PACIENA clinical trial with a historical control group. The pilot clinical trial comprised 7 patients with vaginal agenesis (6 with Rokitansky syndrome and 1 with Morris syndrome) recruited between May 2017 and May 2018 who were operated on at two reference sites. The control group was composed of 9 patients who were operated on at San Juan University Hospital (from 1992 to 2015) using the classical McIndoe technique with the same team of surgeons.

### 2.1. Inclusion Criteria

For cases:

- Women diagnosed with vaginal agenesis due to Rokitansky syndrome or Morris syndrome wishing to undergo neovaginal surgery using the McIndoe technique.
- Adults of legal age and minors with parental authorization.
- A signed informed consent form.

For controls:

- Women diagnosed with vaginal agenesis who were operated on at San Juan University Hospital between 1992 and 2015 using the classical McIndoe technique.

### 2.2. Exclusion Criteria

For cases:

- Women suffering from any medical pathology (metabolic disease, coagulopathy, cardiovascular disease, respiratory disease, Crohn’s disease, rectal disease) that contraindicates surgery or that may worsen the results of neovaginal surgery.
- Minors with no parental authorization.
- The existence of any previous reconstructive neovaginal surgery.

For controls:

- Any other kind of neovaginal surgery undergone other than the McIndoe technique.

### 2.3. Research Plan and Procedures

The data on the control patients were obtained by reviewing the patients’ medical records subsequent to the approval of the San Juan University Hospital Clinical Research Ethics Committee (CEIC number 18/336, approved on 30 October, 2018), and these were registered in a data logbook. The data on the cases were obtained from the SPSS Statistics database created for the publication of the clinical trial.

A new SPSS database with cases and controls was created and this enabled us to compare the results of the two groups.

### 2.4. Outcomes

The main variable in the study was whether or not this surgical procedure really is simpler with improved surgery and hospitalization times, good functional results and patient satisfaction. As such, we analyzed the length of surgery (the length of the intervention starting from the induction of anesthesia) in addition to other parameters in both groups that complemented the main variable, these being:

- The age of the patient at the time of surgery.
- The age of the patient at the time of diagnosis.
- Occurrence of hemorrhage during surgery.
- Occurrence of other incidents during surgery.
- The length of time spent at the hospital.
- The appearance of the neovagina in the speculum assessment (month 1, months 2–4, months 6–12).
- The patient’s symptoms in two periods (months 1–4, months 6–12).
  o Pain from the placement of the prosthesis.
  o Continuous pelvic pain.
  o Foul-smelling discharge.
  o Fever.
- Length of the neovagina at months 1–2.
- The need to change to a smaller maintenance dilator.
- Adequate epithelialization of the vagina.
- The time elapsed until beginning sexual relations.
- Satisfactory sexual relations (at month 4 and month 6).

### 2.5. Statistical Analysis

The statistical analysis was conducted using the SPSS Statistics package, version 25 (IBM, Spain).

A descriptive statistical analysis was conducted using non-parametric tests. Contingency tables and a comparison of proportions were used to determine frequencies and the distribution with qualitative variables. Chi-squared, Kruskal–Wallis and Mann–Whitney U correlation tests were used to compare groups and parameters in the different follow-up procedures.

We conducted non-parametric tests on paired data as a means of comparing the values obtained in each review (months) for quantitative or numerical variables. The Wilcoxon test and the McNemar test were used for dichotomous variables.

A statistically significant difference was included when the *p*-value was <0.05 in all statistical tests.

## 3. Results

Table 1 shows the age of the patient at the time of diagnosis and at the time of surgery, the length of surgery, the time spent in hospital and the length of the neovagina 1–2 months after surgery.

There were no statistically significant differences in the age of the patients at the time of diagnosis and at the time of surgery between the two groups.

**Figure 1:**
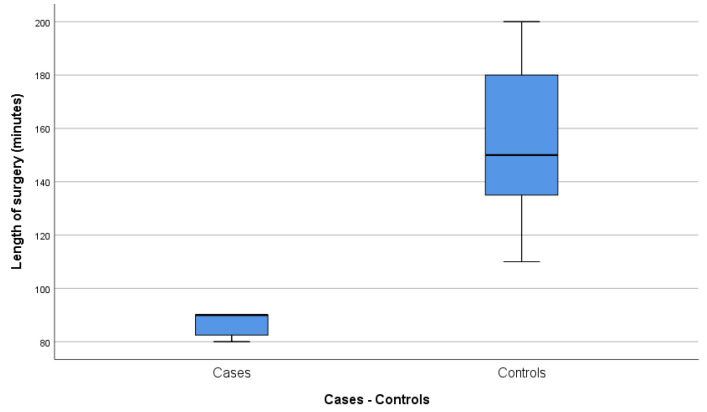
Length of surgery (minutes).

The average length of surgery was 86.43 ± 4.75 (95% CI 82.03–90.83) minutes for the case group and 155.56 ± 28.44 (95% CI 133.69–177.42) minutes for the control group, the difference being statistically significant (*p* < 0.05). In addition, the box diagram (Figure 1) illustrates that the length of most of the surgical procedures in the case group was below average, while it was above average for patients in the control group.

**Figure 2:**
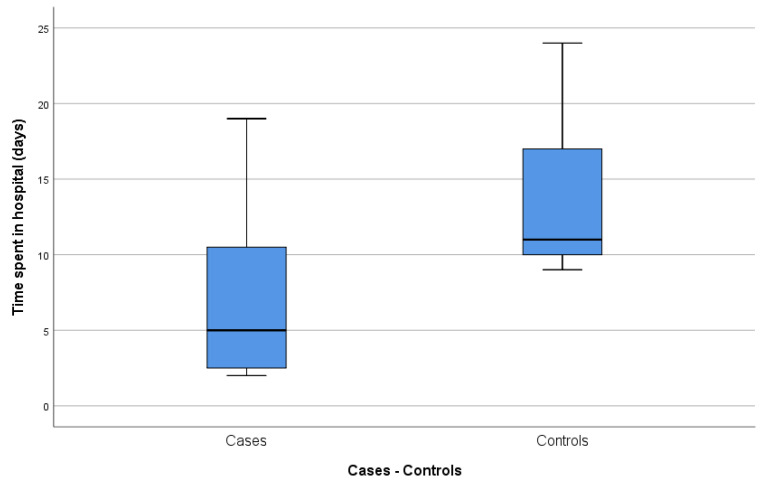
Time spent in the hospital (days).

With regard to the length of hospitalization after surgery (Figure 2), the case group registered an average of 7.43 ± 6.6 (95% CI 1.32–13.54) days, while the average length for the control group was 13.44 ± 5.1 (95% CI 9.52–17.37) days. The case group registered an abnormally large interval due to the fact that the patient with Morris syndrome, who subsequently dropped out of the study, spent a long time in the hospital. For this reason, after eliminating this patient from the database, we obtained an even lower average hospitalization time for the case group, 5.50 ± 4.5 (95% CI 0.68–10.32) days, and a statistically significant difference between both groups (*p* = 0.012).

No differences were found between groups in relation to the amount of bleeding during surgery or in the time that elapsed until the epithelialization of the neovagina. A greater neovaginal length was achieved at 1–2 months after surgery in patients who underwent surgery with no skin graft and a PACIENA^®^ prosthesis, with an average of 8.93 ± 1.42 (95% CI 7.61–10.25) cm, compared with the control group, which registered an average of 6.56 ± 1.13 (95% CI 5.69–7.42) cm. Despite this statistically significant difference (*p* = 0.005) in vaginal length, there were no differences regarding the commencement of sexual relations, or with regard to whether or not the patients encountered any difficulty or dyspareunia during sexual relations, or if these relations were satisfactory.

**Figure 3:**
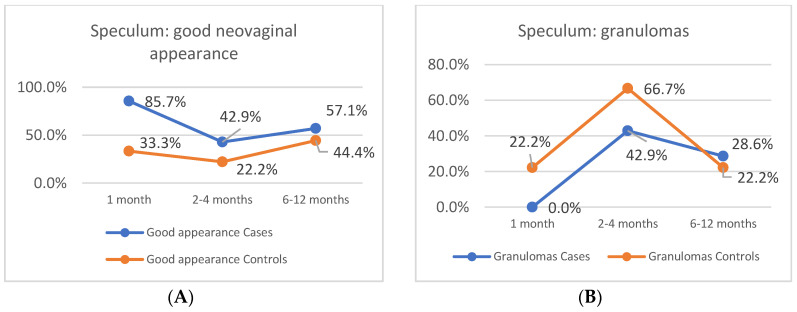
Speculoscopy. (**A**) Percentage of patients with a good neovaginal appearance. (**B**) Percentage of patients with neovaginal granulomas.

With regard to the evolution of the neovagina through speculoscopy, an assessment of the presence of granulomas, infection and foul-smelling discharge was completed, in addition to determination of whether a good appearance was attained. The findings for both groups were grouped into month 1, months 2–4 and months 6–12 visits. The line graph illustrates that the presence of granulomas was lower in the case group in the first two visits with a better neovaginal appearance throughout the follow-up procedure for this group (Figure 3).

To assess the symptoms, we grouped post-surgery visits at months 3–4 and months 6–12. Symptoms (Table 2), such as pain from the placement of the maintenance prosthesis, bleeding and abdominal pain, were included.

## 4. Discussion

Many different techniques to treat vaginal agenesis have been proposed. Apart from the surgical approach, the difference between most neovaginal surgery techniques lies in the material used to line the neovagina; skin grafts, peritoneum and amnion have been described for this purpose. In this sense, new techniques have been developed with other coatings. The use of autologous buccal mucosa is promising and has provided good results but as any other technique with graft intake it implies a greater number of days of admission to prevent complications and the risk of scarring tissue on the donor area [5,6].

The use of a skin graft is justified and cannot be considered a disadvantage when the patient needs a second intervention with skin removal. This is the case of gender confirmation surgery or if the neovaginal procedure is performed with simultaneous abdominoplasty or reduction mammoplasty [7,8,9]. It requires a careful selection of patients, with certain characteristics and with a double surgery indication. However, it inevitably involves a longer surgical time and is not useful for all types of patients.

To avoid skin grafting, Panici et al. [10], in 2007, published a study on the use of autologous vaginal tissue with good results [11,12] but it requires cell culture 2–3 weeks prior to transplantation. Therefore, the major disadvantage of this technique is the cellular therapy component, which means it can only be performed in specialized centers with a cell factory and an operating room available when the tissue is prepared to be transplanted.

Alternatively, and with the same purpose, many authors have described the use of porcine small intestinal submucosa or resorbable meshes, with similar results for neovaginal reconstruction [13]. In 1994, Jackson et al. [14] published the results of surgeries performed on four women with vaginal agenesis who underwent neovaginal surgical construction using an absorbable adhesion mesh (Interceed^®^) to cover an inflatable stent with good anatomical and functional results. The results of a study by Motoyama et al. (2013) [15] involving surgeries performed on 10 patients using a mold coated with Interceed^®^ were also excellent. In that study, correct epithelialization of the neovagina was achieved between 1 and 4 months post-surgery. Inagaki et al. [16], in 2009, also reported two cases that underwent a similar surgical technique consisting of the placement of a cylindrical synthetic resin mold covered with absorbable mesh (Interceed^®^). Zhang et al. [17] registered in 2017 a study on vaginoplasties performed with Interceed^®^ on 40 patients, with the anatomical and sexual functionality results also satisfactory.

The length of surgery in those studies was similar to that patients operated on using the PACIENA^®^ prosthesis technique with no skin graft (86.43 min, on average). The reduction in operative time is explained by avoiding the skin graft step, which leads to a shorter surgery. In our study, there have been no intraoperative complications in either group and blood loss was estimated to be low (<100 mL) in all cases and moderate (100–500 mL) in one of the controls and two patients from the cases group. We also found a reduction in hospitalization time to 5.50 ± 4.5 days in the cases group. Considering that no discharge criteria were created for this study, but general post-surgical criteria were followed, most of the patients with a longer length of admission experienced post-surgical complications; however, none of them were considered significant by the medical team attending the patients, except for the patient with Morris syndrome who presented with postoperative denial and dropped out of the study.

Regardless of the lining of the vagina, all these surgical techniques involve the insertion of a stent into the neovagina to ensure permeability; however, prostheses that are not anatomically correct for the patient have been used. To solve this problem, some authors have proposed the use of molds with different dimensions or personalized molds [18,19]. Anagani et al. [20] recently published data on the modification of the McIndoe technique in 52 patients in India, in which acceptable short-term physiological and psychological results and adequate epithelialization of the neovagina were achieved between 4 and 6 months. An invariable mold was used in these cases. This was created with the third digit of a number eight surgical glove, filled with non-adherent gauze, sealed at the bottom with silk sutures, and covered with antibiotic cream, a condom and two sheets of Interceed^®^ over it.

In contrast, the dimensions and design of the PACIENA^®^ prosthesis adapt to the vagina and the external genital area, providing the patient with comfort. The prothesis is made with polylactic acid (PLA), a biocompatible material whose effect on tissue growth has been studied [21]. In addition, with the maintenance prosthesis, our patients had good anatomical results, including an improvement in vaginal length and no changes with regard to satisfaction in sexual relations, as the 6 cm limit, specified in the literature as being necessary to obtain coital satisfaction, was exceeded in the case group [22].

Although no evidence-based treatment algorithm has been established, the American College of Obstetricians and Gynecologists (ACOG) has been recommending dilation therapy as a first-line approach in the treatment of vaginal agenesis since 2002 [23]; however, the condition can be managed nonsurgically if it is correctly diagnosed and the patient is sufficiently motivated. Effective management includes training in primary vaginal dilation by the gynecologist and a careful psychologic preparation of the patient before any intervention. Patients must know that it will be a slow process, understand it and accept it; otherwise, a surgical technique may be used from the outset. In this regard, Bracaglia et al. [24] proposed to insert a question in the informed consent that allows the evaluation of whether a patient is sufficiently engaged and has completely understood the verbal and written information. All this requires the patience and availability of the surgeon to give all the necessary explanations.

Both in self-dilation and after surgical techniques, it is important to use an appropriate anatomical prosthesis after surgery to maintain vaginal length. In this sense, Herlin et al. [25] published a comparative study in 2018 in which the anatomical results and complications of surgical techniques used for vaginal agenesis were analyzed and compared with non-surgical techniques (self-dilation and coital dilation). There were no statistically significant differences between the vaginal length achieved using the classical McIndoe technique versus that achieved with self-dilation, while there was a higher rate of complications when the surgical technique with a skin graft was used. This once again highlights the importance of using a prosthesis with excellent characteristics to enable us to modify the classical technique.

As such, the combination of the PACIENA^®^ prosthesis, due to its anatomical structure and manufacturing material (PLA), with the anti-adhesion absorbable mesh coating (Interceed^®^) could be successfully used in vaginoplasties, leading us to propose the modification of the classical McIndoe surgical technique. This can maintain good anatomical and functional results and provide an improvement in surgery and hospitalization times.

### 4.1. Clinical Relevance

The need for a neovagina due to vaginal agenesis may be of a congenital nature or may arise from other circumstances such as gender confirmation. Vaginal atresia can result from the post-surgery formation of synechiae, radiotherapy or ablation, among other causes. In any of these situations, the degree to which the patient’s quality of life is affected due to issues regarding body image and sexual factors makes this type of surgical technique extremely important. Therefore, it needs to be developed and improved in order to achieve better results with lower rates of morbidity and mortality.

### 4.2. Study Limitations

The low incidence of diseases associated with vaginal agenesis prevented us from analyzing a larger sample. Some of the records in the historical group of operated patients had to be discarded as the classical technique had not been used. In addition, due to the fact that this is a historical cohort, it was difficult to collect specific data from clinical examinations in control patients who underwent a less homogeneous follow-up procedure.

## 5. Conclusions

Our study illustrates a reduction in surgery and hospitalization times in patients who underwent a surgical technique consisting of a PACIENA^®^ neovaginal prosthesis with no skin graft. These patients achieved good anatomical results, including an improvement in vaginal length and no changes with regard to satisfaction in sexual relations. These results were achieved by comparing two groups of patients with no differences in age at the time of surgery. The results suggest that a modification of the classical McIndoe technique with the use of the aforementioned prosthesis can be recommended.

## Figures and Tables

**Table 1:** Patient data.

Patient	Age at Diagnosis (Year)	Age at Surgery (Year)	Diagnosis	Duration (Minutes)	Time Spent in Hospital (Days)	Neovaginal Length (cm)
CASE 1	13	17	Rokitansky	85	5	9.0
CASE 2	14	19	Rokitansky	90	14	8.0
CASE 3	18	25	Rokitansky	80	2	10.0
CASE 4	18	26	Rokitansky	80	2	11.0
CASE 5	13	19	Rokitansky	90	3	9.0
CASE 6	16	20	Morris	90	19	6.5
CASE 7	16	21	Rokitansky	90	7	9.0
CONTROL 1	15	16	Rokitansky	150	24	7.0
CONTROL 2	17	18	Rokitansky	110	10	7.0
CONTROL 3	16	20	Rokitansky	130	18	7.0
CONTROL 4	16	25	Rokitansky	150	9	8.0
CONTROL 5	16	19	Rokitansky	165	11	6.0
CONTROL 6	16	21	Rokitansky	180	10	6.0
CONTROL 7	16	18	Rokitansky	135	10	4.0
CONTROL 8	17	17	Rokitansky	180	12	7.0
CONTROL 9	14	14	Rokitansky	200	17	7.0

**Table 2:** Symptoms experienced by the case and control groups.

	Cases (*n* = 7)		Controls (*n* = 9)	
Symptoms	3–4 Months	6–12 Months	3–4 Months	6–12 Months
None	3 (42.9%)	5 (71.4%)	5 (55.5%)	6 (66.7%)
Pain placement prothesis	3 (42.9%)	1 (14.3%)	0	0
Bleeding	0	0	3 (33.3%)	0
Abdominal pain	0	0	1 (11.1%)	2 (22.2%)
